# Effects of Three-Layer Encapsulated Tea Tree Oil on Growth Performance, Antioxidant Capacity, and Intestinal Microbiota of Weaned Pigs

**DOI:** 10.3389/fvets.2021.789225

**Published:** 2021-12-02

**Authors:** Lixue Wang, Ying Zhang, Ling Liu, Fei Huang, Bing Dong

**Affiliations:** State Key Laboratory of Animal Nutrition, College of Animal Science and Technology, China Agricultural University, Beijing, China

**Keywords:** tea tree oil, encapsulated, weaned pig, growth performance, antioxidant, intestinal microbiota

## Abstract

Tea tree oil (TTO) exerts key roles in improving growth performance of pigs. However, knowledge is limited regarding comparative effects of Encp TTO and Un-encp TTO supplementation on growth performance of pigs. A study determined the effects of TTO or its capsulation on growth performance, antioxidant capacity, and intestinal microbiome of weaned pigs. A total of 144 healthy pigs (8.5 ± 0.24 kg) were subjected to four treatments for a 28-d trial with six replicates per treatment and six pigs per pen: negative control, NC; positive control, PC (antibiotic supplemented); Un-encp TTO (supplemented with unencapsulated TTO); Encp TTO (supplemented with encapsulated TTO). NC, TTO, and PC treatments were compared with regard to improved average daily gain (ADG), average daily feed intake (ADFI), feed conversion rate, nutrient digestibility, and intestinal morphology (*p* < 0.05) and decreased diarrhea rate. TTO- and PC-treated pigs had higher levels of serum superoxide dismutase, glutathione peroxidase, and immunoglobulin G; lower levels of liver aspartate aminotransferase and alanine aminotransferase; and improved concentrations of interleukin 10 (IL-10), tumor necrosis factor α, and IL-1β (*p* < 0.05). TTO- and PC-treated pigs had higher abundance of beneficial bacterial species *Subdoligranulum* and lower abundance of diarrhea associated species *Escherichia–Shigella* in cecal and colonic digesta (*p* < 0.05). Encapsulation of TTO preserved more activities of TTO than its unencapsulated counterpart by showing higher ADG, ADFI, and feed conversion rate during day 1 (d1) to d14 (*p* < 0.05) and tended to lower diarrhea rate (*p* = 0.083) and improve villous height/crypt depth (VH/CD) ratio (*p* = 0.089) in jejunum. Encapsulation of TTO also improved antioxidant indexes and decreased liver injury and inflammation accordingly (*p* < 0.05). Encapsulated TTO-treated pigs had higher abundance of beneficial *Clostridium_sensu_stricto_1* and lower the abundance of harmful *Escherichia–Shigella* in cecal and colonic digesta (*p* < 0.05). Our results demonstrated TTO benefits on improving growth performance of weaned pigs and further proved that encapsulation of TTO was superior to its unencapsulated counterpart at multiples. Encapsulated TTO was similar to the PC group and could be potentially an alternative of feed antibiotics for weaned pigs.

## Introduction

Under weaning stress, piglets often experience diarrhea, digestion dysfunction, low utilization rate of feed, suppressed immune response, and changed composition of intestinal microbes (1). Supplementing diets with antibiotics is one of the main means to reduce diarrhea and promote growth in newly weaned piglets. However, routine use of antibiotics may pose a significant threat to human health (2). Consequently, many scientists seek to develop novel alternatives to antibiotics. Essential oils are a potential alternative as they are residue-free and broad-spectrum and are generally recognized as safe and beneficial (3, 4).

Tea tree oil (TTO) is an essential oil steam-distilled from *Melaleuca alternifolia* (5). Tea is widely planted in tropical and subtropical areas of China (6). Tea oil production reached 2.61 million metric tons in 2019 in China (7). TTO contains more than 100 compounds, including α-pinene, terpene-4-ol, linalool, and α-terpenol, which have antibacterial activities (8). Dietary TTO exhibited prophylactic effects on weaned pigs by improving the immune response and growth performance (9–11).

Use of TTO as a feed additive presents many challenges. TTO is unstable and can be damaged by environmental factors such as oxygen, heat, light, and humidity when stored (12). It has unstable functional groups in which oxygen acts as an oxidizing agent for alcohols and aldehydes, light radiation changes unsaturated monoterpenic and sesquiterpenic hydrocarbons, and water plays a role in ether and ester hydrolysis (13). For example, Rudback et al. (14) demonstrated that α-terpinene, an antioxidant in TTO, autoxidizes rapidly to allergens on exposure to air. TTO is a lipophilic liquid that contains many volatile compounds, such as alcohols, ethers, and esters in TTO, which produce a smell that may affect the palatability of diets for piglets (8). However, the effects of TTO on feed intake of weaned pigs were inconsistent, ranging from 3% depression to 19% increase (15).

New encapsulation of TTO is able to overcome above challenges by coating particles or droplets in a matrix to give a physical barrier between the core compound and the surroundings. Therefore, encapsulation protects them from environmental damage and controls their release (16–18). We hypothesized that supplemental encapsulation of TTO might exert positive effects on feed intake, and may have better effects on growth performance, immune response, and intestinal microflora of weaned pigs compared with unencapsulated TTO.

To the best of our knowledge, no studies have specifically reported the effective difference introduced by encapsulation process on TTO activity in weaned pigs. Therefore, the aim of the present study was to (1) evaluate the effects of TTO (regardless of encapsulation) on growth performance, diarrhea rate, immune response, antioxidative activity, and bacteria composition in gastrointestinal tract of weaned pigs; (2) compare the different effect between encapsulated and unencapsulated TTO on the above indexes in weaned pigs.

## Materials and Methods

This experiment was carried out at the National Feed Engineering Technology Research Center at the Ministry of Agriculture Feed Industry Center Animal Testing Base (Hebei, China). All procedures used in this study were conducted in accordance with Chinese Guidelines for Animal Welfare and approved by the China Agricultural University Institutional Animal Care and Use Committee (201905610420678).

### Preparation of Encapsulated TTO

**Figure 1:**
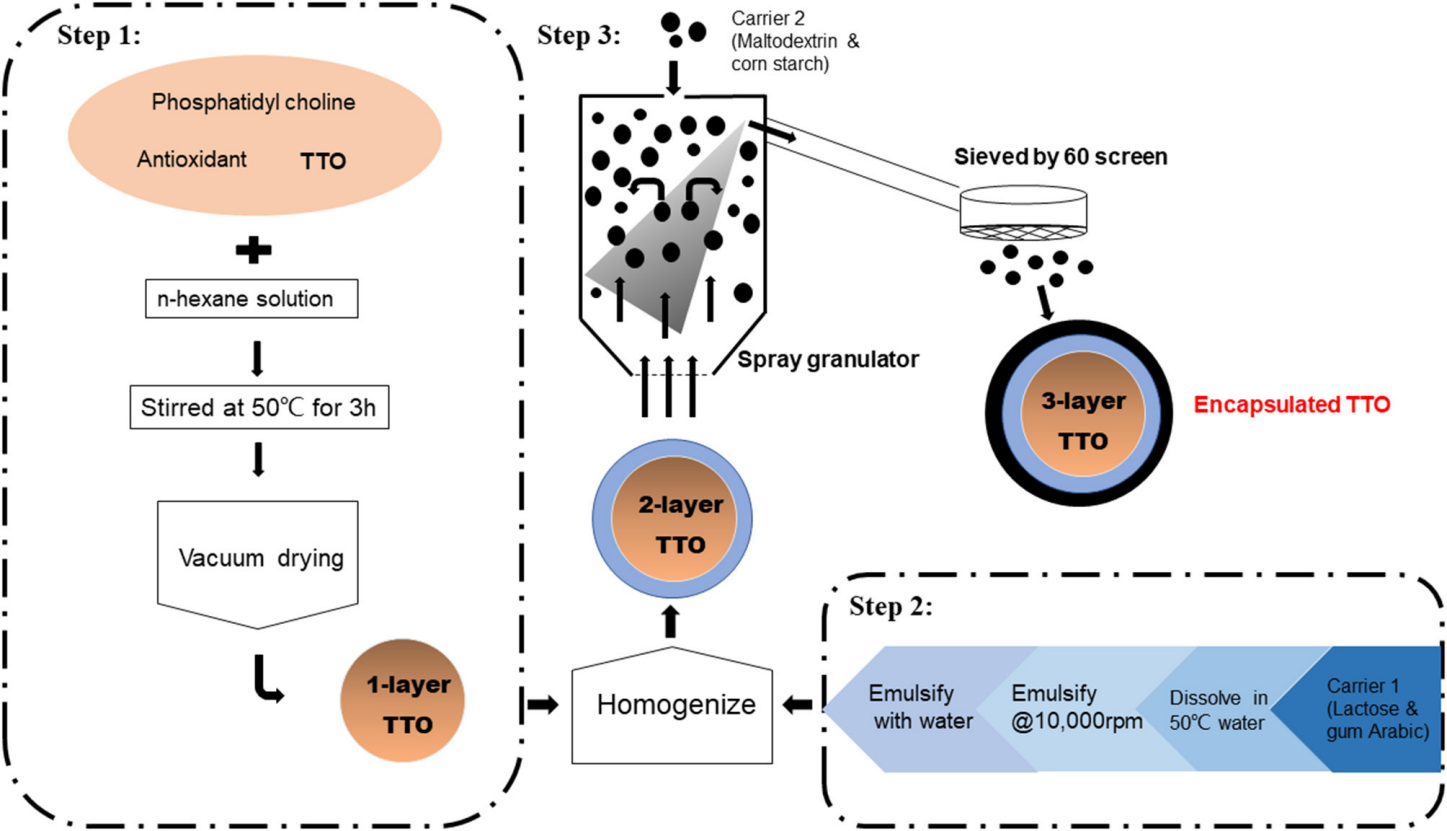
Preparation of encapsulated TTO.

Commercial TTO (1 kg) was mixed with phosphatidyl choline (1 kg) isolated from soybean, and an antioxidant (0.2 kg vitamin E) in n-hexane solution (2 L; Figure 1; Hebei Chenguang Biotech Group Co., Ltd.). The solution was heated to 50°C and stirred for 3 h followed by vacuum drying to form 1 layer on TTO particles. Carrier material was prepared by mixing lactose (4 kg) and gum arabic (3.75 kg) in water (25 L, 50°C). This carrier solution was emulsified at 9,600 g. Dried 1-layer TTO particles were mixed with the carrier solution and further emulsified at 9,600 for 30 min and homogenized at a pressure of 20 to 60 MPa for two to three times. To this second step, two-layer encapsulated TTO was produced. Finally, another carrier, mixture of maltodextrin and cornstarch (1:2 wt/wt), was spray-dried on two-layer TTO and sieved (60-mesh screen) forming encapsulated three-layer TTO particles. The diameter of the one-layer particle composed of TTO and phosphatides was from 2 to 20 μm, and the diameter of the three-layer encapsulated TTO was from 75 to 250 μm.

### Gas Chromatography–Mass Spectrometry Analysis of TTO

Commercial TTO was obtained from Hebei Chenguang Biotech Group Co., Ltd. (Handan, China), and extraction was performed by the steam distillation technique, utilizing leaves of tea trees. The analysis was conducted using gas chromatography–mass spectrometry (QP2010, Shimadzu Company, Japan) according to Adams (19). The ionization voltage, mass range, and the condition of GC condition were set according to previously described methods (20). Peaks were identified that corresponded to the data from mass spectra library (National Institute of Standard and Technology). Finally, quantitative determination was conducted based on the peak area integration.

### Animals, Diets, and Experimental Design

Piglets (*n* = 144, Duroc × Landrace × Large white, weaned at 28 days after birth) with an average initial weight of 8.5 ± 0.24 kg were used in this experiment. This study designed six replicates per treatment with six piglets (three male and three female piglets) per pen, and the experiment was separated into phase 1 (days 1–14) and phase 2 (days 15–28). Dietary treatments included basal diet (NC), basal diet supplemented with 75 mg/kg aureomycin (PC), basal diet supplemented with 0.4% unencapsulated TTO (Un-encp TTO; unencapsulated TTO was sprayed on porous starch and dried at 20°C for 30–50 min), and basal diet supplemented with 0.4% encapsulated TTO (Encp TTO; the encapsulation strategy was described in “preparation of encapsulated TTO”). The actual content of TTO in each product was 11% (Hebei Chenguang Biotech Group Co., Ltd.). We added 0.4% encapsulated or unencapsulated TTO to the basic diet, so the actual value of TTO among treatments was 0.04%. All diets were formulated to meet or exceed the requirements suggested by National Research Council (NRC, 2012) estimates of the nutrient requirements of weaned piglets (Table 1). Chromic oxide (0.3%) was added to each treatment diet as an indigestible marker to calculate the apparent total tract digestibility (ATTD) of nutrients. Pigs were housed in pens that measured 1.2 ×2.1 m and were equipped with stainless-steel feeder and one nipple waterer with slatted plastic floors. The environmental temperature was controlled and maintained at 24–26°C. Pigs had *ad libitum* access to feed and water.

**Table 1:** Composition and nutrient levels of the basal diets (%, as-fed basis)^a^.

	**Diet**	
**Items**	**Phase 1 (days 0–14)**	**Phase 2 (days 15–28)**
**Ingredient, %**		
Corn	59.52	64.09
Soybean meal	15.00	15.80
Extruded soybean	6.3	6.0
Fish meal	4.0	3.5
Whey powder	4.0	3.15
Soy protein concentrate	4.8	2.8
Soybean oil	2.2	1.12
Dicalcium phosphate	1.15	1.0
Limestone	0.82	0.6
NaCl	0.3	0.3
l-Lysine HCl, 78.8%	0.52	0.44
dl-Methionine, 98.5%	0.18	0.13
l-Threonine, 98.5%	0.18	0.14
l-Tryptophan, 98.5%	0.03	0.03
Chromic oxide	0.3	0.30
Choline chloride	0.2	0.10
Premix^b^	0.5	0.50
Total	100	100
**Nutrient levels, calculated^c^**		
Digestible energy, kcal/kg	3,540	3,490
Crude protein	20.79	19.52
SID lysine	1.39	1.25
Sid methionine	0.49	0.47
Methionine + cystine	0.76	0.79
Calcium	0.84	0.70
Phosphorus	0.65	0.61
**Mycotoxin level, measured^d^**		
DON, μg/kg	1,738.82	1,526.97
ZEA, μg/kg	240.47	253.16
AFB1, μg/kg	4.74	5.39

a *Experimental diets were a corn–soybean meal–based diet (NC), NC + 75 mg/kg aureomycin (PC), NC + 0.4% unencapsulated TTO (Un-encp TTO), and NC + 0.4% encapsulated TTO (Encp TTO)*.

b *The premix provided the following per kilogram of the diet: vitamin A, 12,000 IU; vitamin D_3_, 2,500 IU; vitamin E, 30 IU; vitamin K_3_, 30 mg; vitamin B_12_, 12 μg; vitamin B_2_, 4 mg; vitamin B_5_, 15 mg; niacin, 40 mg; choline chloride,400 mg; folic acid, 0.7 mg; vitamin B_1_, 1.5 mg; vitamin B_6_, 3 mg; biotin, 0.1 mg; Mn, 40 mg; Fe, 90 mg; Zn, 100 mg; Cu, 8.8 mg; I, 0.35 mg; Se, 0.3 mg*.

c *Values were calculated according to NRC (2012)*.

d *Values were measured by UPLC-MS/MS analysis*.

*AFB1, aflatoxin B1; DON, deoxynivalenol; SID, standardized ileal digestible; ZEA, zearalenone*.

Each piglet was weighed on days 0, 14, and 28, and feed consumption was also recorded on a pen basis to determine average daily gain (ADG), average daily feed intake (ADFI) and the feed to gain ratio (F:G). Fecal consistency was assessed visually three times per day by observers blinded to treatments according to previously described methods (21). The scoring system was applied to determine the rate of diarrhea as following: 0 = firm and normally shaped, 1 = soft (retained some shape), 2 = brown liquid, 3 = frequent passage of watery feces (22). The occurrence of diarrhea was defined as a continuous 2-day fecal score at level 2 or 3. Diarrhea rate was calculated using the following formula. Diarrhea rate (%) = (number of piglets with diarrhea × diarrhea days) / (number of piglets × total observational days) ×100%.

### Sample Collection

On the morning of days 14 and 28, one pig closest to the average body weight in each pen was selected to collect blood samples (8 mL) via the anterior vena cava puncture into vacuum tubes following a 12-h fasting. Serum samples were separated by centrifugation at 3,000 g at 4°C for 15 min and stored at −20°C until further analysis.

On the morning of day 28, six pigs per treatment group were randomly selected and were euthanized by electric shock and then bled. The selected pigs were from different pens, and their body weights were close to the average body weight of pigs in each pen. The small intestine was dissected from the mesentery and immediately placed on ice. Segments (2 cm in length) of the middle parts of the duodenum, jejunum, and ileum without any digesta were fixed in 10% neutral-buffered formalin for subsequent morphological examination. Liver tissues were collected and quickly washed with ice-cold saline solution and then homogenized in ice-cold phosphate buffer (phosphate-buffered saline: 1:9 wt/vol, pH 7.4) and centrifuged at 2,000 g for 20 min. The supernatant was collected and stored at −80°C for inflammatory cytokine analysis. Liver microsomes were prepared by differential centrifugation (11,000 and 2 ×100,000 g) in a Tris/KCl buffer (20 mM, pH 7.4) and stored at −80°C for cytochrome P450 (P450) analysis (23).

Fecal samples for determination of nutrient digestibility were collected from each pen during the last 3 days (days 25–27) and were immediately stored at −20°C. At the end of the experiment, the 3-day collection of fecal samples was pooled by pen and then dried in a forced-air drying oven at 65°C for 72 h. Feed and dried fecal samples were ground finely to pass through a 1-mm screen (40-mesh) before chemical analysis. For analysis of gut microbiota, digesta in the cecum and colon of slaughter piglets were sampled (each sample from one pig per pen) in sterile tubes on day 28 and immediately immersed in liquid nitrogen and then stored at −80°C (24).

### Apparent Total Tract Digestibility of Feed Nutrients

Dry matter (DM; method 934.01), crude protein (CP; method 990.03), and ether extract (EE; method 920.39) of feed and fecal samples were determined according to the Association of Official Analytical Chemists (25). Neutral detergent fiber (NDF) concentration of these samples were determined according to the method of McCarthy et al. (26). The content of chromium in feed and fecal samples was determined by atomic absorption spectrophotometer (Hitachi Z-5000, Tokyo, Japan) (27).

### Serum Antioxidant, Immune, and Biochemical Parameter Analysis

In the serum, concentrations of glutathione peroxidase (GSH-Px) and superoxide dismutase (SOD) were determined according to the manufacturer's instructions using commercial kits (Jiancheng Biochemical Reagent Co., Nanjing, China). The content of serum immunoglobulins A, M, and G (IgA, IgM, and IgG) were measured using commercially available enzyme-linked immunosorbent assay (ELISA) kits following manufacturer's instructions (Nanjing Jiancheng Bioengineering Institute, Nanjing, China). Serum biochemical parameters including aspartate aminotransferase (AST), alanine aminotransferase (ALT), and alkaline phosphatase (ALP) were measured using corresponding commercially available kits (BioSino Bio-technology and Science Inc., Beijing, China).

### Cytokine Measurement and Enzyme Assays

In serum and liver, concentrations of interleukin 10 (IL-10), tumor necrosis factor α (TNF-α), and IL-1β were determined using the corresponding commercially available ELISA kits (Beijing Sino-UK Institute of Biological Technology, Beijing, China). Concentrations of the microsomal cytochromes P450 were determined using the spectrophotometric method of Omura and Sato (28).

### Intestinal Morphology

Intestinal morphology was characterized according to the methods of our previous study (22). Briefly, fixed intestinal samples were dehydrated and embedded in paraffin. Embedded samples were cut into slices (6 μm thick) and stained with hematoxylin-eosin. Then six villous height (VH, determined as the distance between the crypt opening to the end of the villous), crypt depth (CD, measured from the crypt–villous junction to the base of the crypt), and VH/CD (VH/CD) ratio were measured under a light microscope (Nikon, Tokyo, Japan) at a 40 × magnification and were analyzed using Image-Pro Plus 6.0 software.

### Microbiota Analysis

Microbial DNA was extracted from cecal and colonic digesta using the E.Z.N.A.® stool DNA kit (Omega Bio-tek, Norcross, GA, USA) according to the manufacturer's instructions. Markers and adaptors-linked universal primers 338F (ACTCCTACGGGAGGCAGCAG) and 806R (GGACTACHVGGGTWTCTAAT) targeting the V3–V4 region were used to amplify microbial 16S rRNA. The polymerase chain reaction (PCR) amplification of 16S rRNA gene was performed as follows: 95°C for 3 min, followed by 25 cycles at 95°C for 30 s, 55°C for 30 s, and 72°C for 45 s and a final extension at 72°C for 10 min (29). The PCR product was extracted with 2% agarose gel and purified using the AxyPrep DNA Gel Extraction Kit (Axygen Biosciences, Union City, CA, USA). Purified amplicons were pooled in equimolar proportions and paired-end sequenced (2 ×300) on an Illumina MiSeq platform (Illumina, San Diego, CA, USA). The processing of sequencing data was conducted as previously described (30). The data presented in the study are deposited in the NCBI repository, accession number PRJNA677843. Operational taxonomic units (OTUs) with a 97% similarity cutoff (31) were clustered using UPARSE (version 7.1, http://drive5.com/uparse/), and chimeric sequences were identified and removed. Taxonomy of each OTU representative sequence was analyzed by RDP Classifier (http://rdp.cme.msu.edu/) against the 16S rRNA database (Silva SSU128) using confidence threshold of 0.7.

### Statistical Analysis

GLM procedure of SAS (SAS Institute Inc., Cary, NC, USA) was used for data analysis. Each pen of pigs was considered as an experimental unit. Diarrhea rates were analyzed by χ^2^ test (SAS Institute Inc., Cary, NC, USA). Planned orthogonal contrasts were used to evaluate the overall effect of TTO ([NC] vs. [Un-encp TTO, Encp TTO]) or encapsulation effect ([Un-encp TTO] vs. [Encp TTO]). All data were checked for normal distribution and homogeneity of variance using the Shapiro–Wilk normality test and Bartlett test, respectively. The differences and composition of bacterial abundance were analyzed by Kruskal–Wallis rank-sum test (29). *p* < 0.05 was considered statistically significant, and 0.05 ≤ *p* < 0.10 was indicative of a differential trend.

## Results

### Composition of TTO

The total yield of volatile compounds from encapsulated TTO was 2.74% ± 0.22%. Fourteen compounds from the encapsulated TTO were detected by gas chromatography–mass spectrometry (Table 2). Two volatile compounds, terpinen-4-ol (50.31%) and γ-terpinene (20.06%), accounted for the majority of volatiles measured.

**Table 2:** Volatile compounds detected from encapsulated TTO.

**Components**	**Content (%)**
Terpinen-4-Ol	50.31
γ-Terpinene	20.06
α-Terpinene	5.28
Terpinolene	4.59
α-Terpineol	3.05
α-Pinene	3.32
ρ-Cymene	0.58
1,8-Cineole	0.58
Aromadendrene	0.5
δ-Cadinene	0.42
Ledene	0.35
Limonene	0.17
Sabinene	0.15
Globulol	0.1

### Effect of TTO on Growth Performance and Diarrhea Rate

Regardless of encapsulation, compared with the negative control treatment, TTO and positive control treatment increased (*p* < 0.05) ADG at phase 1 (days 0–14), phase 2 (days 15–28), and overall (days 0–28) and increased (*p* < 0.05) ADFI at phase 2 and overall (Table 3). TTO and PC treatment decreased F:G (*p* < 0.01) and diarrhea rate (*p* < 0.01) compared with the NC treatment. In addition, there were no differences in growth performance between TTO treatment and PC treatment. Concerning the encapsulation effect, compared with the Un-encp TTO treatment, the treatment of Encp TTO had higher ADG at phase 1, phase 2, and overall phases (*p* < 0.05) and higher ADFI in phase 2 and overall phases (*p* < 0.05). Encp TTO treatment had lower F:G at phase 1 (*p* < 0.05) and tended to have lower diarrhea rate compared with Un-encp TTO treatment (*p* = 0.083).

**Table 3:** Effects of TTO and encapsulation on growth performance and diarrhea rate of weaned pigs (*n* = 6)^a^.

**Item**	**PC**	**NC**	**Un-Encp TTO**	**Encp TTO**	**SEM**	***p* value**	**Contrast** ***p*** **value**	
							**TTO effect^b^**	**Encapsulation effect^c^**
**BW, kg**								
0 d	8.6	8.4	8.5	8.4	0.244	0.354	0.231	0.553
14 d^d^^,^ ^e^	13.6	12.9	13.2	13.4	0.368	<0.001	0.001	0.075
28 d^d^^,^ ^e^^,^ ^f^	22.0	20.2	21.1	21.7	0.770	<0.001	<0.001	0.003
**0**–**14 d**								
ADG, g/d^d^^,^ ^e^^,^ ^f^	357	321	336	357	19.982	<0.001	0.005	0.006
ADFI, g/d^d^	543	508	514	522	19.971	0.006	0.213	0.470
F:G^e^^,^ ^f^	1.52	1.58	1.53	1.46	0.084	0.076	0.037	0.012
**15**–**28 d**								
ADG, g/d^d^^,^ ^e^^,^ ^f^	600	521	564	593	35.885	<0.001	<0.001	0.045
ADFI, g/d^d^^,^ ^e^^,^ ^f^	972	902	942	972	36.159	<0.001	0.001	0.026
F:G^d^^,^ ^e^	1.62	1.73	1.68	1.64	0.070	0.010	0.018	0.418
**0**–**28 d**								
ADG, g/d^d^^,^ ^e^^,^ ^f^	479	421	450	475	25.281	<0.001	<0.001	0.003
ADFI, g/d^d^^,^ ^e^^,^ ^f^	747	690	707	736	27.612	<0.001	0.007	0.022
F:G^d^^,^ ^e^	1.56	1.64	1.57	1.55	0.065	0.020	0.024	0.687
**Diarrhea rate, %**								
0–28 d^d^^,^ ^e^	3.15	6.02	4.2	3.19	1.459	<0.001	<0.001	0.083

a *Experimental diets were a corn–soybean meal–based diet (NC), NC + 75 mg/kg aureomycin (PC), NC + 0.4% unencapsulated TTO (Un-encp TTO), and NC + 0.4% encapsulated TTO (Encp TTO)*.

b *Orthogonal contrast statement: [NC] versus [Un-encp TTO, Encp TTO]*.

c *Orthogonal contrast statement: [Un-encp TTO] versus [Encp TTO]*.

d *Letter represents statistical difference among dietary treatments (p <0.05)*.

e *Letter represents statistical difference between [NC] and [Un-encp TTO, Encp TTO] (p <0.05)*.

f *Letter represents statistical difference between [Un-encp TTO]and [Encp TTO] (p <0.05)*.

*A trend is declared when p is no more than 0.10 (0.05 ≤ p ≤ 0.1)*.

*BW, body weight; ADG, average daily gain; ADFI, average daily feed intake; F:G, feed-to-gain ratio*.

### Effect of TTO on Apparent Total Tract Digestibility of Nutrient

TTO treatment (Un-encp TTO and Encp TTO) increased ATTD of CP, EE, and DM compared with the negative control treatment (*p* < 0.05, Table 4). TTO treatment tended to increase ATTD of EE (*p* = 0.089) compared with the PC treatment. There were no differences in ATTD of DM, CP, and NDF between TTO treatment and PC treatment. Encapsulation of TTO (Encp TTO vs. Un-encp TTO) had no significant effect on ATTD of these nutrients.

**Table 4:** Effects of TTO and encapsulation on nutrient digestibility of weaned pigs (*n* = 6)^a^.

**Item**	**PC**	**NC**	**Un-encp TTO**	**Encp TTO**	**SEM**	***p* value**	**Contrast** ***p*** **value**	
							**TTO effect^b^**	**Encapsulation effect^c^**
DM, %^d^^,^ ^e^	86.73	85.24	87.00	87.19	0.969	0.021	0.019	0.748
CP, %^d^^,^ ^e^	81.80	79.19	82.95	83.15	1.806	0.002	0.003	0.798
EE, %^e^	67.81	66.58	69.55	70.75	2.277	0.089	0.038	0.384
NDF, %	61.02	60.67	61.95	61.56	1.216	0.643	0.515	0.717

a *Experimental diets were a corn–soybean meal–based diet (NC), NC + 75 mg/kg aureomycin (PC), NC + 0.4% unencapsulated TTO (Un-encp TTO), and NC + 0.4% encapsulated TTO (Encp TTO)*.

b *Orthogonal contrast statement: [NC] vs. [Un-encp TTO, Encp TTO]*.

c *Orthogonal contrast statement: [Un-encp TTO] vs. [Encp TTO]*.

d *Letter represents statistical difference among dietary treatments (p <0.05)*.

e *Letter represents statistical difference between [NC] and [Un-encp TTO, Encp TTO] (p <0.05)*.

*A trend is declared when p is no more than 0.10 (0.05 ≤ p ≤ 0.1)*.

*CP, crude protein; EE, ether extract; NDF, nitrogen-free extract; DM, dry matter*.

### Effect of TTO on Small Intestinal Morphology

Pigs fed the TTO and positive control diets had higher VH and VH/CD ratio in the jejunum than those fed NC diet (*p* < 0.05, Table 5). The VH, CD, and VH/CD ratio in the duodenum and ileum did not differ among NC, PC, and TTO treatments (Un-encp TTO and Encp TTO). Encp TTO tended to increase the VH/CD ratio (*p* = 0.089) in the jejunum compared with the Un-encp TTO treatment.

**Table 5:** Effects of TTO and encapsulation on small intestinal morphology of weaned pigs (*n* = 6)^a^.

**Item**	**PC**	**NC**	**Un-encp TTO**	**Encp TTO**	**SEM**	***p* value**	**Contrast** ***p*** **value**	
							**TTO effect^b^**	**Encapsulation effect^c^**
**Duodenum**								
VH (μm)	336.25	320.45	341.29	346.23	18.098	0.368	0.246	0.753
CD (μm)	264.81	261.16	267.79	270.63	9.144	0.684	0.489	0.717
VH/CD	1.27	1.23	1.27	1.28	0.082	0.887	0.695	0.927
**Jejunum**								
VH (μm) ^d^^,^ ^e^	349.28	312.42	351.85	365.62	25.300	0.028	0.019	0.337
CD (μm)	240.88	260.24	264.55	240.54	17.902	0.218	0.239	0.145
VH /CD^d^^,^ ^e^	1.45	1.20	1.33	1.52	0.158	0.027	0.017	0.089
**Ileum**								
VH (μm)	353.45	321.31	345.28	367.10	26.128	0.176	0.130	0.342
CD (μm)	248.91	246.83	253.88	254.93	9.675	0.734	0.601	0.905
VH/CD	1.42	1.31	1.36	1.44	0.108	0.461	0.384	0.354

a *Experimental diets were a corn–soybean meal–based diet (NC), NC + 75 mg/kg aureomycin (PC), NC + 0.4% unencapsulated TTO (Un-encp TTO), and NC + 0.4% encapsulated TTO (Encp TTO)*.

b *Orthogonal contrast statement: [NC] vs. [Un-encp TTO, Encp TTO]*.

c *Orthogonal contrast statement: [Un-encp TTO] vs. [Encp TTO]*.

d *Letter represents statistical difference among dietary treatments (p <0.05)*.

e *Letter represents statistical difference between [NC] and [Un-encp TTO, Encp TTO] (p <0.05)*.

*A trend is declared when p is no more than 0.10 (0.05 ≤ p ≤ 0.1)*.

*VH, villous; CD, crypt depth*.

### Effect of TTO on Antioxidant and Immunoglobulin Indexes in Serum

Serum SOD, GSH-Px, and IgG in piglets fed TTO and PC diets were higher than the NC treatment (*p* < 0.05, Table 6). Serum IgG was greater in pigs fed the TTO diet than those fed the PC diet. Compared with the Un-encp TTO and PC treatment, piglets fed with Encp TTO showed higher serum SOD and GSH-Px activities (*p* < 0.05).

**Table 6:** Effects of TTO and encapsulation on serum antioxidant and immune indexes of weaned pigs (*n* = 6)^a^.

**Item**	**PC**	**NC**	**Un-encp TTO**	**Encp TTO**	**SEM**	***p* value**	**Contrast** ***p*** **value**	
							**TTO effect^b^**	**Encapsulation effect^c^**
SOD, U/mL^d^^,^ ^e^^,^ ^f^	146.45	141.25	147.90	155.74	5.812	0.001	0.012	0.010
GSH-Px, U/mL^d^^,^ ^e^^,^ ^f^	750.76	714.69	758.81	805.70	37.128	0.002	0.011	0.032
IgA, g/L	1.01	0.94	1.03	1.05	0.078	0.299	0.220	0.756
IgM, g/L	0.76	0.77	0.76	0.78	0.061	0.967	0.899	0.681
IgG, g/L^d^^,^ ^e^	7.76	7.76	9.26	9.88	1.027	<0.001	0.002	0.172

a *Experimental diets were a corn–soybean meal–based diet (NC), NC + 75 mg/kg aureomycin (PC), NC + 0.4% unencapsulated TTO (Un-encp TTO), and NC + 0.4% encapsulated TTO (Encp TTO)*.

b *Orthogonal contrast statement: [NC] vs. [Un-encp TTO, Encp TTO]*.

c *Orthogonal contrast statement: [Un-encp TTO] vs. [Encp TTO]*.

d *Letter represents statistical difference among dietary treatments (p <0.05)*.

e *Letter represents statistical difference between [NC] and [Un-encp TTO, Encp TTO] (p <0.05)*.

f *Letter represents statistical difference between [Un-encp TTO]and [Encp TTO] (p <0.05)*.

*A trend is declared when p is no more than 0.10 (0.05 ≤ p ≤ 0.1)*.

*IgA, immunoglobulin A; IgM, immunoglobulin M; IgG, immunoglobulin G; SOD, superoxide dismutase; GSH-Px, glutathione peroxidase*.

### Effect of TTO on Serum Enzyme Activities and Inflammatory Cytokines of Weaned Pigs

Pigs fed TTO exhibited lower serum concentrations of ALT and AST compared with pigs fed the NC and PC treatments (*p* < 0.05, Table 7). In addition, supplementation with TTO increased serum level of IL-10 (*p* < 0.05) and tended to decrease the level of TNF-α (*p* > 0.05) compared with NC and PC treatments. Compared with pigs fed Un-encp TTO and PC, Encp TTO decreased concentrations of AST and increased concentration higher level of IL-10 (*p* < 0.05).

**Table 7:** Effects of TTO and encapsulation on serum enzyme activities and inflammatory cytokines of weaned pigs (n = 6)^a^.

**Item**	**PC**	**NC**	**Un-encp TTO**	**Encp TTO**	**SEM**	***p* value**	**Contrast** ***p*** **value**	
							**TTO effect^b^**	**Encapsulation effect^c^**
ALT, U/L^d^^,^ ^e^	24.47	23.24	20.65	19.05	2.571	0.009	0.024	0.273
AST, U/L^d^^,^ ^e^^,^ ^f^	39.49	37.05	35.66	32.60	2.662	<0.001	0.028	0.006
ALP U/L	281.29	271.83	285.32	305.24	18.602	0.149	0.114	0.214
TNF-α, pg/mL	65.23	73.26	69.33	64.11	4.875	0.052	0.062	0.204
IL-1β, pg/mL^d^	34.65	36.79	38.11	39.46	2.226	0.019	0.111	0.375
IL-10, pg/mL^d^^,^ ^e^^,^ ^f^	16.36	16.04	17.35	18.76	1.298	0.002	0.012	0.049

a *Experimental diets were a corn–soybean meal–based diet (NC), NC + 75 mg/kg aureomycin (PC), NC + 0.4% unencapsulated TTO (Un-encp TTO), and NC + 0.4% encapsulated TTO (Encp TTO)*.

b *Orthogonal contrast statement: [NC] vs. [Un-encp TTO, Encp TTO]*.

c *Orthogonal contrast statement: [Un-encp TTO] vs. [Encp TTO]*.

d *Letter represents statistical difference among dietary treatments (p <0.05)*.

e *Letter represents statistical difference between [NC] and [Un-encp TTO, Encp TTO] (p <0.05)*.

f *Letter represents statistical difference between [Un-encp TTO]and [Encp TTO] (p <0.05)*.

*A trend is declared when p is no more than 0.10 (0.05 ≤ p ≤ 0.1)*.

*ALT, alanine aminotransferase; AST, aspartate aminotransferase; ALP, alkaline phosphatase; TNF-α, tumor necrosis factor; IL-1β, interleukin 1β; IL-10, interleukin 10*.

### Effect of TTO on Inflammatory Cytokines and P450 in Liver of Weaned Pigs

**Figure 2:**
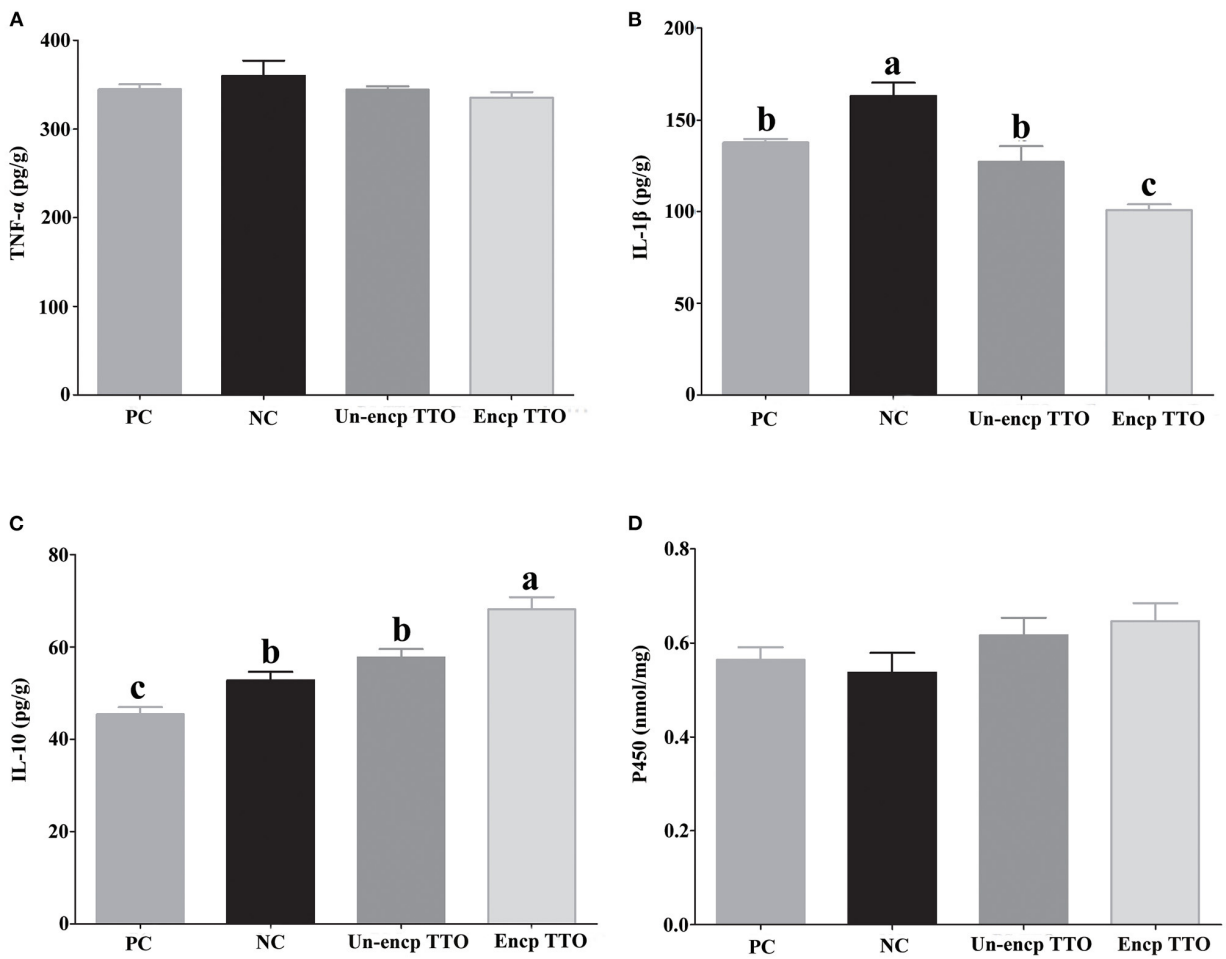
Expression of tumor necrosis factor α (TNF-α), interleukin 1β (IL-1β), and interleukin 10 (IL-10), contents of cytochrome P450 (P450) in livers of weaned pigs (*n* = 6). Experimental diets were control diet (NC), NC + 75 mg/kg aureomycin (PC), NC + NC + 0.4% unencapsulated TTO (Un-encp TTO), and NC + 0.4% encapsulated TTO (Encp TTO). Values are means, error bars indicate SEM, and different letters above a bar denote the significant difference between treatments (*p* < 0.05). **(A)** Relative expression of TNF-α. **(B)** Relative expression of IL-1β. **(C)** Relative expression of IL-10. **(D)** Contents of P450. Statistical analysis was performed using one-way ANOVA.

Pigs fed TTO treatments had lower levels of IL-1β (*p* = 0.004) in the liver than those in the negative control treatment (Figures 2B,C). Compared with the Un-encp TTO and positive control treatments, lower level of IL-1β and higher level of IL-10 were observed in pigs fed Encp TTO (*p* < 0.05). There were no significant differences in concentration of TNF-α and P450 in liver among experimental treatments (Figures 2A,D).

### Effect of TTO on Intestinal Microbiota Composition

**Figure 3:**
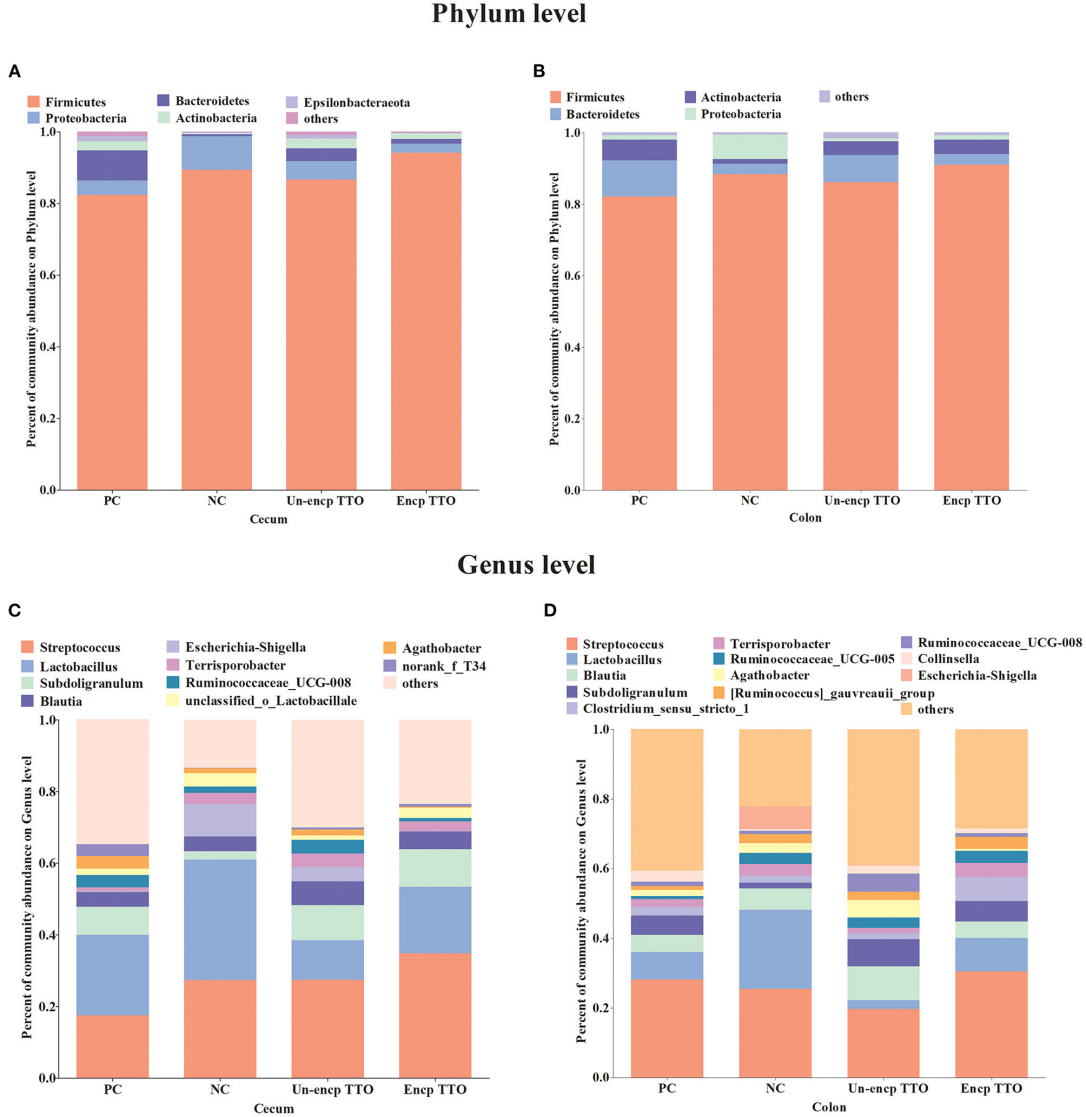
Effects of dietary treatment on intestinal bacterial communities of weaned pigs (*n* = 5). Bacterial taxa present at relative abundances of >0.01% and the proportion of other bacteria is <0.01% at the phylum level, whereas at the genus level, it was 0.03%. Experimental diets were control diet (NC), NC + 75 mg/kg aureomycin (PC), NC + 0.4% unencapsulated TTO (Un-encp TTO), and NC + 0.4% encapsulated TTO (Encp TTO). **(A)** Community barplot analysis for the cecal bacterial communities at the phylum level. **(B)** Community barplot analysis for the colonic bacterial communities at the phylum level. **(C)** Community barplot analysis for the cecal bacterial communities at the genus level. **(D)** Community barplot analysis for the colonic bacterial communities at the genus level.

**Figure 4:**
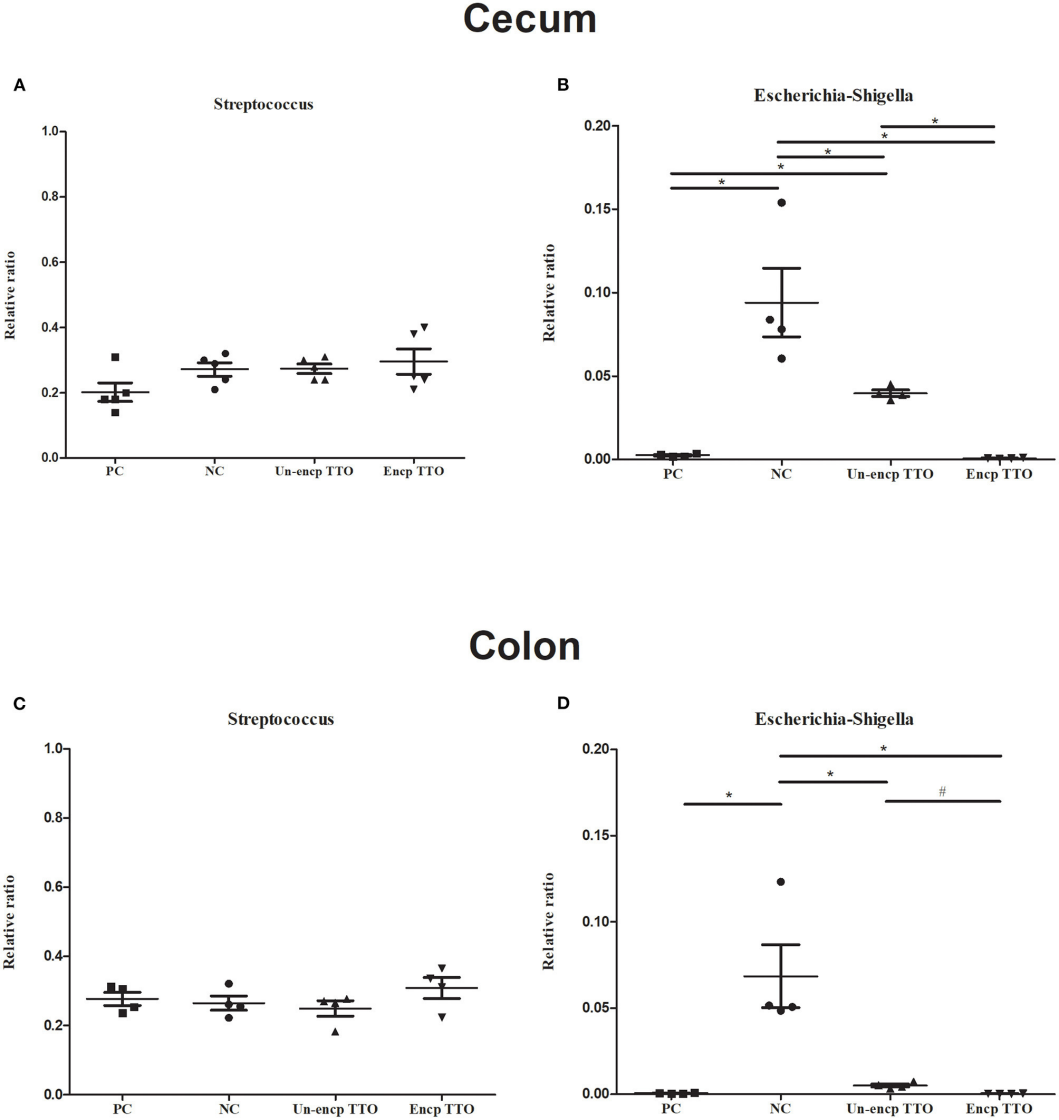
Effects of diet treatment on the relative abundance of *Escherichia–Shigella* and *Streptococcus* of weaned pigs at the genus level (*n* = 5). Experimental diets were control diet (NC), NC + 75 mg/kg aureomycin (PC), NC + 0.4% unencapsulated TTO (Un-encp TTO), and NC + 0.4% encapsulated TTO (Encp TTO). **(A)** The relative abundance of cecal *Streptococcus*. **(B)** The relative abundance of cecal *Escherichia–Shigella*. **(C)** The relative abundance of colonic *Streptococcus*. **(D)** The relative abundance of colonic *Escherichia–Shigella*. Error bars represent standard error of the mean. The *p* values are based on Kruskal–Wallis tests. *^,#^Differences were considered significant at *p* < 0.05 when compared with NC, Un-encp TTO groups, respectively.

We observed a shift in the microbial composition of cecal and colonic digesta (Figure 3). The dominant bacteria phyla across treatments were mainly Firmicutes, Bacteroidetes, and Proteobacteria. At the genus level, abundance of *Subdoligranulum* in cecal and colonic microbial communities in the TTO (Un-encp TTO and Encp TTO) and PC treatments was higher than that in the NC treatment (Figures 3C,D), whereas *Escherichia–Shigella* were lower (*p* < 0.05, Figures 4B,D). Compared with the Un-encp TTO and PC treatments, pigs fed the Encp TTO diets had higher relative abundance of *Clostridium_sensu_stricto_1* in the colonic digesta (Figure 3D) and lower relative abundance of *Escherichia–Shigella* (Figure 4B) in the cecal and colonic digesta (*p* < 0.05). Moreover, the relative abundance of *Streptococcus* in cecum and colon was not altered among all treatments (Figures 4A,C).

## Discussion

TTO comprises multiple identified constituents including terpinene, terpinolene, and terpineol, which account for more than 85% of the total mixture. Terpinen-4-ol accounts for nearly 60% of the major components of TTO. Because of the antimicrobial activity of its components (32–34), TTO exhibits *in vitro* antimicrobial activity (35). In the current study, we revealed TTO *in vivo* may be a potential alternative to antibiotics in weanling pigs. In addition, we further investigated the mechanisms of TTO function.

Our observation that TTO increased ADG and improved feed conversion rate is consistent with previously reported studies of TTO (9, 10) and terpinene in an essential oil blend (36). The positive effect on ADG and feed conversion rate may be related to the increase in feed intake promoted by TTO. TTO likely contributed to elevated feed intake by two mechanisms. Many types of essential oils emit appealing smells to arouse pigs' interest in feed, whereas other essential oils emit aversive smells that may deter piglets from feed intake [reviewed by Zeng (15)]. TTO appeared to positively stimulate pig appetite as evidenced by the increased feed intake of pigs over the experimental period. Encapsulated TTO induced even higher feed intake, which previously hid TTO smells. This phenomenon indicated the proper coating process of TTO that favored animal sensory. Feed intake is regulated by postingestional signals such as released gastrointestinal hormones or stimulus from gastrointestinal nerves (37). Higher feed intake indicates an expressed well-being process of digestion (38). Encapsulation process of TTO preserved its activity from being oxidized and volatizing to air. Higher activity of encapsulated TTO during digestion by improving nutrient utilization contributed to benefit feed intake.

TTO increased digestibility of protein, fat, and DM in diets. These results are consistent with feeding other essential oils to pigs (9, 39–41) and broilers (42). The above essential oils are similar to TTO and contain numerous monoterpenes and sesquiterpene compounds. Essential oils stimulate secretion of saliva and bile, leading to elevated activity of digestive enzymes (43). High monoterpene present in essential oils has spasmolytic effect to increase the interaction between feed and digestive enzymes (44). Improved VH and villous-to-crypt ratio in the jejunum may have led to higher absorption of digested nutrients, which contributed to increased ADG of weanling piglets. Improved morphology was associated with higher nutrient digestibility in previously reported studies (45).

In the current study, TTO showed increased antioxidant potential reflected by higher levels of serum SOD and GSH-Px compared with unsupplemented control and positive control pigs. TTO also enhanced the anti-inflammatory effect represented by higher levels of IL-10 in serum compared with NC- and PC-fed pigs. In addition, TTO treatment enhanced IgG level in serum compared with NC- and PC-fed pigs. For many types of essential oils, enhanced antioxidant potential often occurs concurrently with greater anti-inflammation and greater immunoglobulin levels in serum (46–48). The systemically beneficial effects of TTO helped protect the liver from injury, which was marked by lower levels of serum ALT and ASP. In a study of uninfected animals, TTO did not stimulate immune responses (49). When rats and sheep were infected by bacteria or gastrointestinal parasites, TTO helped protect the animals by activating cellular and humoral immune responses and decreasing levels of ALT and ASP (50, 51). Previous study observed that *Escherichia coli* challenge caused oxidative injury when compared with the unchallenged pigs (52). In the current study, TTO impressively inhibited abundance of *E. coli–Shigella* in cecum and colon. Meanwhile, we found three mycotoxins (DON, ZEA, and AFB1) present in the experimental diets of this study. Animals that ingest mycotoxin-contaminated diets exhibit reduced feed intake and growth rate (53, 54). Mycotoxins also promoted inflammatory injury of livers in piglets (55–57). Mycotoxins are produced by fungi such as *Aspergillus, Fusarium, Penicillium*, and *Alternaria* (58, 59). These fungal genera are found commonly in crops and cereals, especially when storage conditions are humid and temperature is not well controlled (60), leading to contaminated feed [reviewed by Pitt et al. (61)]. In China, mycotoxins in feed are widely present (62). Terpinene-4-ol and gamma-terpinene, active components in TTO, can inhibit growth of toxic fungi *in vitro* and thus inhibit production of mycotoxins (35, 63). In this study, Encp TTO was prepared with antioxidant, which helped it maintain more antioxidant activity of TTO and explain why Encp TTO exhibited better effects on antioxidation and anti-inflammation compared with its unencapsulated counterpart.

Diarrhea in weaned piglets causes severe economic loss globally. Infections are mainly colibacillosis diarrhea (64). In China, especially in warm and rural areas, the infection rate in weaned piglets was 25%, and the death rate reached 21% (65). Weaning is a critical period for piglets when piglets encounter multiple stressors such as changes of diets, social interactions, and environment (66). These stressors usually damage intestinal structures, making pigs more susceptible to infection, and induce unbalanced gut microbiota (67). *In vitro*, TTO has antibacterial activity by disrupting the microbial cell membrane which leads to loss of chemiosmotic control (68, 69). In the current *in vivo* study, TTO impressively inhibited abundance of *E. coli–Shigella* in cecum and colon, and lowered diarrhea rate of weanling piglets was comparable to that of PC supplementation in diets. *Shigella* are taxonomically part of *E. coli* species characterized by invasion of colonic and rectal mucosa, which provokes a strong inflammatory response and destruction of colonic epithelium (70). Signs of shigellosis include severe, bloody diarrhea and mucous in feces. *Shigella* are the principal bacterial cause of sustained endemic diarrhea (71). *Shigella* species have developed antibiotic resistance in the last 20 years (72–74). In this respect, TTO has potential to control *Shigella*-caused diarrhea in weanling piglets. When comparing the effective difference between unencapsulated TTO and encapsulated TTO, we found that encapsulated TTO reduced *Shigella* abundance in the cecum and in the colon, where *Shigella* specifically invade. This is another example that supports the notion that encapsulation preserves more activities of TTO from loss of function in digestion and cross-link reactions in gastrointestinal tract.

On the phylum level, TTO treatment led to higher counts of dominant phyla (Firmicutes and Bacteroidetes) and fewer Proteobacteria than in NC treatment. The bacteria of Firmicutes and Bacteroidetes in gut microbiota can ferment carbohydrates to a variety of short-chain fatty acids (SCFAs), and strengthen intestinal barrier functions (75, 76). An increased proportion of Proteobacteria is a potential signature of microbial dysbiosis and risk of intestinal diseases (77). On the genus level, TTO treatment caused higher relative abundance of *Subdoligranulum* and *Clostridium_sensu_stricto_1* in digesta than NC treatment. *Clostridium_sensu_stricto* is reported to produce SCFAs and promote the intestinal mucus barrier (78, 79). *Subdoligranulum* has anti-inflammatory activity and plays a key role in human colon health as a butyrate-producing bacterium (80–82). These results demonstrate that TTO (especially encapsulated TTO) reduces diarrhea rate by reducing pathogenic *E. coli* and improving the abundance of beneficial bacteria in weanling pigs.

## Conclusions

TTO (regardless of encapsulation) improved growth performance, diarrhea rate, immune responses, antioxidative activity, and bacterial composition in gastrointestinal tract of weaned piglets. Encapsulation of TTO enhanced TTO effects on the above indexes. Besides, encapsulated TTO significantly reduced diarrhea rate and reduced the abundance of *E. coli–Shigella* in colon and cecum, which were similar to the antibiotic treatment. This result indicated encapsulated TTO as a potential alternative of feed antibiotics for weaned pigs.

## Data Availability Statement

The datasets presented in this study can be found in online repositories. The names of the repository/repositories and accession number(s) can be found below: PRJNA677843.

## Ethics Statement

The animal study was reviewed and approved by Chinese Guidelines for Animal Welfare and approved by the China Agricultural University Institutional Animal Care and Use Committee. Written informed consent was obtained from the owners for the participation of their animals in this study.

## Author Contributions

LW and BD designed and wrote the manuscript. YZ and LL critically edited the text. LW, LL, and BD finalized the manuscript. All authors read and approved the final manuscript.

## Conflict of Interest

The authors declare that the research was conducted in the absence of any commercial or financial relationships that could be construed as a potential conflict of interest.

## Publisher's Note

All claims expressed in this article are solely those of the authors and do not necessarily represent those of their affiliated organizations, or those of the publisher, the editors and the reviewers. Any product that may be evaluated in this article, or claim that may be made by its manufacturer, is not guaranteed or endorsed by the publisher.
